# Ocular Disorders in Adult Leukemia Patients in Nigeria

**DOI:** 10.4103/0974-9233.63081

**Published:** 2010

**Authors:** Afekhide E. Omoti, Caroline E. Omoti, Rita O. Momoh

**Affiliations:** Department of Ophthalmology, University of Benin Teaching Hospital, PMB 1111, Benin City, Edo State, Nigeria; 1 Department of Haematology, University of Benin Teaching Hospital, PMB 1111, Benin City, Edo State, Nigeria

**Keywords:** Leukemia, Ocular Disorders, Proptosis, Retinal Detachment

## Abstract

**Context:**

Leukemias may present with, or be associated with ocular disorders.

**Aims:**

To determine the rates of ophthalmic disorders in adult patients with leukemia.

**Settings and Design:**

A prospective study of ocular disorders in adult patients with leukemia at the University of Benin Teaching Hospital, Benin City, Nigeria, between July 2004 and June 2008 was conducted.

**Methods and Materials:**

The patients were interviewed and examined by the authors and the ocular findings were recorded. Statistical analysis was performed using Instat GraphPad™ v2.05a statistical package software. The means, standard deviation, and the Kruskal-Wallis non parametric test were performed.

**Results:**

Forty-seven patients with leukemias were seen. Nineteen patients (40.4%) had CLL, 14(29.8%) had CML, 9(19.1%) had AML and 5(10.6%) had ALL. Seven patients (14.9%) had ocular disorders due to leukemia. The ocular disorders due to the leukemia were proptosis in two patients (4.3%), retinopathy in one patient (2.1%), conjunctival infiltration in one patient (2.1%), periorbital edema in one patient (2.1%), retinal detachment in one patient (2.1%), and subconjunctival hemorrhage in one patient (2.1%). There was no significant difference in rate of the ocular disorders in the various types of leukemia (Kruskal-Wallis KW= 4.019; corrected for ties. P=0.2595). One patient (2.1%) was blind from bilateral exudative retinal detachment while 1 patient (2.1%) had monocular blindness from mature cataract.

**Conclusions:**

Ophthalmic disorders that are potentially blinding occur in leukemias. Ophthalmic evaluation is needed in these patients for early identification and treatment of blinding conditions.

## INTRODUCTION

Leukemias are diffuse clonal neoplastic disorders of the bone marrow and blood cells.^1^ They are classified into myeloid or lymphoid based on their origin and into acute and chronic depending on the clinical course. The chronic leukemias arise from the lymphocytic (chronic lymphocytic leukemia, CLL) or myeloid (chronic myelocytic leukemia, CML) precursor cells. They are different from acute leukemias in that the morphology of the cell lines show marked differentiation and are divided into acute lymphocytic leukemia (ALL) and acute myelocytic leukemia (AML). Leukemias may present with, or be associated with ocular disorders. Ocular disorders have been reported in 30-90% of cases of leukemia.^2^–^6^ Orbital and ocular lesions have also been reported to be the third most frequent extramedullary location of acute leukemias after the meninges and testicles.^3^ As a high proportion of patients now achieve initial bone marrow remission with combination chemotherapy, it is important to take a closer look at sites of extramedullary leukemic infiltration, both because of their local morbidity and that these sites may act as a reservoir for proliferation of leukemic cells which may eventually result in systemic relapse.^7^ Furthermore, improved survival of patients with leukemia has led to an increase in variability of ocular presentations in the form of side effects of the treatment and the ways leukemic relapses are being first identified as an ocular presentation.^8^ Late presentation in advanced stages of disease is common in African patients.^9^ The pattern of ocular disease in African patients with hematological malignancies may thus be different from that reported in studies from more advanced countries. This study was designed to identify the common ocular manifestations of leukemia in adult African eyes.

## MATERIALS AND METHODS

All new adult patients with a diagnosis of any of the leukemias attending the Hemato-oncology clinics or admitted into the medical wards of the University of Benin Teaching Hospital, Benin City, Nigeria, between the month of July 2004 and June 2008 were evaluated in the eye clinic of the hospital. Diagnosis was established based on clinical information and cytological features of well-stained (Leishman) peripheral blood smears and bone marrow aspiration cytology. The hematological parameters such as the complete blood count were done using an automated Coulter Counter.

Exclusion criteria included patients who had received chemotherapy, patients on follow-up and children below the age of 17 years. All the new adult patients were interviewed by the authors using a standard questionnaire and were examined in the eye clinic. The visual acuity was determined using the standard illuminated Snellen's chart or illiterate E chart held at 6 m from the patient and the presenting visual acuity recorded. When the patient could not read the chart, the ability to count fingers at varying distances to perceive hand movements or light was determined and recorded. Near vision was tested using the Jaeger's reading chart. Ophthalmic examination was done using a flashlight, Haag Streit slit-lamp biomicroscope and the direct and indirect ophthalmoscopy.. Fluorescein staining of corneal lesions was done whenever indicated. The intraocular pressures were measured using the Goldmann applanation tonometer. Visual field analysis was done using the Kowa automatic visual field plotter (Kowa Company Limited, Okayama, Japan). Refraction was performed in those patients whose visual acuity improved using a pinhole. The best corrected visual acuity was then used to define blindness. Blindness was defined using the World Health Organization definition.^10^ Visual impairment was defined as best corrected vision less than 6/18. Ethical approval for this study was obtained from the Ethical Committee of the University of Benin Teaching Hospital, Benin City, Nigeria.

Data obtained from this study was analyzed by computer with the aid of the Instat GraphPad v2.05a statistical package software. An initial frequency count and percentages were obtained for all the data. The range, mean and standard deviation were determined for the ages of the patients. Relevant data was displayed in tabular form. The standard error of the mean (SEM) and the 95% confidence intervals (CI) were determined and test for statistical significance was calculated using the Kruskal–Wallis non parametric ANOVA test and a p value less than 0.05 was considered to be significant.

## RESULTS

A total of 94 eyes of 47 patients with leukemias were examined during the study period. There were 32 males (68.1%) and 15 females (31.9%) giving a male to female ratio of 2.13: 1. The age range was 20 years to 70 years with a mean of 40.63 years (SD±14.7). The SEM was 7.34 and the 95% CI was 23.26-57.98 years. Nineteen patients (40.4%) had CLL, 14(29.8%) had CML, 9(19.1%) mad AML and 5(10.6%) had ALL.

Twenty-seven patients (57.4%) complained of ocular symptoms while 20 patients (42.6%) had no ocular symptoms. The most common ocular symptoms were poor vision, difficulty in reading, red eyes, itching, eye pain and headache. Some of the patients had multiple ocular complaints [Table 1]. Seven patients (14.9%) had ocular symptoms due to leukemia. The most common ocular disorders found on examination of the patients were refractive errors/presbyopia, age-related macular degeneration, cataract, allergic conjunctivitis, proptosis, and glaucoma [Table 2]. Some patients had more than one ocular disorder. The ocular disorders that may be directly related to the leukemia included one case of leukemia-related retinopathy (2.1%) and one case of retinal detachment (2.1%) causing poor vision, two cases of proptosis (4.3%) resulting in protruding eyes, one case of conjunctival infiltration by leukemia cells (2.1%) and one case of subconjunctival hemorrhage (2.1%) causing red eyes, while the one case of lid swelling was due to periorbital edema (2.1%). Both of the patients with proptosis had bilateral proptosis. One occurred in AML and the other which was more severe and associated with bilateral exudative retinal detachment occurred in a 20-year-old male patient with CML. Retinopathy occurred in a 61-year-old male patient with AML who had retinal hemorrhages, retinal edema, optic disc edema, macular edema and angioid streak. There was no significant difference in the rate of ocular disorders between the various types of leukemia (Kruskal–Wallis; KW= 4.019; corrected for ties. *P* = 0.2595).

**Table 1:** Ocular symptoms in patients with leukemia

Ocular symptoms	Number of patients (%)
Poor vision	20 (42.6)
Difficulty reading	12 (25.5)
Red eye	6 (12.8)
Itching	4 (8.5)
Pain	3 (6.4)
Headache	3 (6.4)]
Protruding eyes	2 (4.3)
Grittiness	2 (4.3)
Something growing over the black of the eye (pterygium)	1 (2.1)
Lid swelling	1 (2.1)

**Table 2:** Ocular disorders in adult patients with leukemia.

Ocular disorders	ALL	AML	CML	CLL	Total
	No (%)	No (%)	No (%)	No (%)	No (%)
Refractive error	1 (20)	2 (22.2)	3 (21.4)	4 (21.1)	10 (21.3)
Presbyopia	0 (0)	1 (11.1)	4 (28.6)	7 (36.8)	12 (25.5)
Age-related macula degeneration	0 (0)	0 (0)	2 (14.3)	3 (15.8)	5 (10.6)
Cataract	0 (0)	0 (0)	1 (7.1)	2 (10.5)	3 (6.4)
Allergic conjunctivitis	1 (20)	1 (11.1)	0 (0)	0 (0)	2 (4.3)
Glaucoma	0 (0)	0 (0)	1 (7.1)	1 (5.3)	2 (4.3)
*Proptosis	0 (0)	1 (11.1)	1 (7.1)	0 (0)	2 (4.3)
*Retinopathies	0 (0)	1 (11.1)	0 (0)	0 (0)	1 (2.1)
*Conjunctival infiltration	0 (0)	1 (11.1)	0 (0)	0 (0)	1 (2.1)
*Periorbital edema	0 (0)	0 (0)	0 (0)	1 (5.3)	1 (2.1)
Pterygium	1 (20)	0 (0)	0 (0)	0 (0)	1 (2.1)
*Subconjunctival hemorrage	0 (0)	1 (11.1)	0 (0)	0 (0)	1 (2.1)
*Retinal detachment	0 (0)	0 (0)	1 (7.1)	0 (0)	1 (2.1)

* Ocular disorders due to leukemia

The two eyes of one patient (2.1%) were blind from bilateral exudative retinal detachment due to metastatic infiltration by leukaemia while another eye (2.1%) was blind from mature cataract. Four patients (8.5%) were visually impaired [Table 3]. The causes of visual impairment were cataract, retinopathy, proptosis and age-related macular degeneration.

**Table 3:** Visual acuities in eyes of adult patients with leukemia.

Visual acuity	Better eye (%)	Worse eye (%)
6/4-6/9	32 (68.1)	25 (53.2)
6/12-6/18	10 (21.3)	11 (23.4)
6/24-CF 3metres	4 (8.5)	9 (19.1)
<CF3m-NLP	1 (2.1)	2 (4.3)
Total	47 (100)	47 (100)

CF- Counting fingers, NLP- No light perception

## DISCUSSION

Late presentation of patients with hematological malignancies is a challenge in Nigeria, and has been documented in previous studies from the same institution.^9^,^11^ Ocular disorders may be the presenting symptoms or may complicate already diagnosed hematological malignancies.^2^–^6^ Ocular disorders are relatively common in leukemias.^2^–^7^ In a study of 288 cases of leukemia, eye changes were seen more often in adults (49.1%) than in children (16.5%), and in myeloid leukemia (41.0%) than in lymphoid leukemia (29.2%).^4^ In this report, ocular symptoms were present in 57.4% of the patients. However, several symptoms and ocular disorders identified on ocular examination were not related to the hematological malignancy. These included refractive errors, presbyopia, cataract, allergic conjunctivitis, age-related macular degeneration and pterygium. Cases of poor vision were mainly due to refractive errors and cataract. Redness, itching and grittiness were due to allergic conjunctivitis and cases of eye growth were due to pterygium. These are conditions which are common in the general population and not related to leukemia or therapy. In a similar study performed in Ethiopia, a variety of miscellaneous ocular findings, such as cataract, pterygium, pingecula, were detected in 36% of all leukemia patients.^5^ These findings indicate the importance of a complete ophthalmologic evaluation in the diagnosis, follow-up and management of leukemia patients.

The conditions that were directly related to the leukemia included proptosis, retinopathy, conjunctival infiltration by malignant tissue, periorbital edema, subconjunctival hemorrhage, and retinal detachment. The lesions were regarded as leukemia-related after excluding other possible causes and in the case of conjunctival infiltration and proptosis, histological diagnosis was also obtained. The total number of cases with ocular involvement due to leukemia was 7 (14.9%) which is less than the 30-90% reported in studies from the diaspora.^2^–^6^ This may be because only new patients at presentation were included in this study and the small numbers of patients seen. The low incidence of ocular disorders in leukemia may also represent a racial difference but more extensive studies are required to make a definitive conclusion. In a prospective study of 74 newly diagnosed Ethiopian patients with leukemia, primary ocular involvement was detected in 32% of cases.^5^ Furthermore, some of the studies that report a very high incidence may have included disorders which may not necessarily be related to leukemia.^5^ Ocular complications of leukemia are reported to be more severe in patients with high-risk leukemia than in patients with standard risk leukemia.^12^

We found a higher incidence of males in this study which is in agreement with another study that reported male preponderance.^13^ However, this may be a manifestation of the increased likelihood of males to utilize health services in sub-Saharan Africa. The most common specific ocular manifestation of leukemia in this report was proptosis which was mainly due to infiltration of the orbital tissues by malignant cells.^1^ A case of CML presented with exudative retinal detachment in both eyes secondary to tumor infiltration. This was the only case of bilateral blindness in this report. Retinal detachment has also been reported in CLL in other studies.^14^,^15^ The ocular changes result either from a direct effect of metastatic neoplastic infiltration or compression or by circulating antibodies involving paraneoplastic retinal degeneration. Infiltration and effect of circulating antibodies may be responsible for the retinopathies and retinal detachment.^1^

The incidence of bilateral blindness and monocular blindness was low, albeit several potentially blinding conditions were observed that included retinal detachment, cataract, orbital disease, retinopathies, age-related macular degeneration, and glaucoma. The cases of glaucoma in this report did not significantly impair the vision of the affected patients and were only discovered during the study. These conditions are either treatable or preventable, but early diagnosis is required in most of the cases to prevent blindness.

In conclusion, ocular morbidity occurs in adult patients with leukemia. Several of these are potentially blinding conditions which may be related or unrelated to the disease. Early ophthalmic evaluation is recommended to identify and treat these conditions.
